# The role of gaping behaviour in habitat partitioning between coexisting intertidal mussels

**DOI:** 10.1186/1472-6785-10-17

**Published:** 2010-07-12

**Authors:** Katy R Nicastro, Gerardo I Zardi, Christopher D McQuaid, Linda Stephens, Sarah Radloff, Gregory L Blatch

**Affiliations:** 1 CCMAR, CIMAR-Laboratorio Associado, Universidade do Algarve, Gambelas, 8005-139, Faro, Portugal; 2 Department of Zoology & Entomology, Rhodes University, Grahamstown 6140, South Africa; 3 Department of Biochemistry, Microbiology & Biotechnology, Rhodes University, Grahamstown 6140, South Africa; 4 Department of Statistics, Rhodes University, Grahamstown 6140, South Africa

## Abstract

**Background:**

Environmental heterogeneity plays a major role in invasion and coexistence dynamics. Habitat segregation between introduced species and their native competitors is usually described in terms of different physiological and behavioural abilities. However little attention has been paid to the effects of behaviour in habitat partitioning among invertebrates, partially because their behavioural repertoires, especially marine benthic taxa, are extremely limited. This study investigates the effect of gaping behaviour on habitat segregation of the two dominant mussel species living in South Africa, the invasive *Mytilus galloprovincialis *and the indigenous *Perna perna*. These two species show partial habitat segregation on the south coast of South Africa, the lower and upper areas of the mussel zone are dominated by *P. perna *and *M. galloprovincialis *respectively, with overlap in the middle zone. During emergence, intertidal mussels will either keep the valves closed, minimizing water loss and undergoing anaerobic metabolism, or will periodically open the valves maintaining a more efficient aerobic metabolism but increasing the risk of desiccation.

**Results:**

Our results show that, when air exposed, the two species adopt clearly different behaviours. *M. galloprovincialis *keeps the shell valves closed, while *P. perna *periodically gapes. Gaping behaviour increased water loss in the indigenous species, and consequently the risk of desiccation. The indigenous species expressed significantly higher levels of stress protein (Hsp70) than *M. galloprovincialis *under field conditions and suffered significantly higher mortality rates when exposed to air in the laboratory. In general, no intra-specific differences were observed in relation to intertidal height. The absence of gaping minimises water loss but exposes the invasive species to other stresses, probably related to anoxic respiration.

**Conclusions:**

Gaping affects tolerance to desiccation, thus influencing the vertical zonation of the two species. Valve closure exposes the invasive species to higher stress and associated energy demands, but it minimizes water loss, allowing this species to dominate the upper mussel zone, where the gaping indigenous *P. perna *cannot survive. Thus even very simple behaviour can influence the outcome of interactions between indigenous and invasive species.

## Background

To define the dynamics of species coexistence, it is necessary to understand how different species respond to environmental stress at a physiological, ecological and also a behavioural level [1,2]. This is particularly important when investigating interactions among indigenous and invasive species. By interacting with the diverse biological attributes of species, environmental heterogeneity plays a defining role in invasion and coexistence dynamics [3,4]. As both indigenous species and invaders respond to environmental variations, it is the difference in their responses that determines the success of the invader and how it interacts with native species [5,6]. Habitat segregation among species is usually described in terms of competition [7-9]) and of different physiological properties or responses of species [10-12]. While, reports about the role of behaviour in habitat partitioning among vertebrates are relatively common (e.g. [13-15]), they are rare for both terrestrial and marine invertebrates (but see [16-18]). For many invertebrates, behaviour involves minimal movement carried out over comparatively long time periods and at small spatial scales. However, such behaviour may be critical for survival and its quantification may provide crucial insights into species distributions [19].

Coastal ecosystems are under particularly heavy threat [20] from invasive species and organisms living in the intertidal are subjected to highly variable and often extreme environmental conditions [21]. They are regularly covered and uncovered by the movement of tides that subject them to a transition from aquatic to terrestrial conditions. Therefore distribution and vertical zonation of intertidal communities are affected by the exceptionally steep gradients in temperature, desiccation and oxygen availability that are typical of this habitat [21-24]. This is particularly true for sessile and sedentary organisms that are not capable of moving to evade environmental stresses imposed during low tide exposure to air (emersion). Several studies have shown that life in the high intertidal involves adaptation responses such as increased thermal resistance [25], heat stability of key metabolic enzymes [26], increased extracorporeal water storage, reduced evaporation [25,27] and stress-induced expression of heat stress proteins (Hsps; [28]). Hsps are molecular chaperones that prevent aggregation of damaged proteins and facilitate their renaturation following stress [29]. Previous studies have shown that Hsps induction is related to heat and hypoxic stress in several organisms, and their detection and quantification have become a sensitive and rapid mode of screening for stress responses (e.g. [30]).

Intertidal bivalves are important ecological engineers on rocky shores, providing habitat for many species of infauna and epifauna. During emersion, they either close their valves and undergo anaerobic metabolism, or exhibit alternate closure and opening of the shell (gaping), allowing the maintenance of aerobic respiration [31,32]. The first behaviour leads to reduced evaporative water loss at the cost of inefficient exploitation of organic energy; gaping is more compatible with optimal functioning of the metabolic machinery, but at the same time valve movements push water out of the mantle cavity, increasing levels of water loss and the risk of desiccation [33,34]. Despite the increase in water loss, gaping does not appear to have a significant influence on body temperature through evaporative cooling for the species examined to date [19,35-37], and it seems most likely that gaping occurs as a means of increasing aerobic respiration rather than evaporative cooling [17,38,35].

The Mediterranean mussel *Mytilus galloprovincialis *is one of the most widespread marine invasive species worldwide and is now globally distributed throughout the temperate zones of the northern and southern hemispheres [39-41]. It is believed to have been introduced to South Africa in the late 1970 s [42]. It has subsequently spread along the entire west coast of the country, extending into Namibia and has more recently spread for 800-900 km along the south coast as far as East London [43]. On the south coast, it shows partial habitat segregation with the indigenous *Perna perna *in the lower eulittoral zone (referred to here as mussel zone). *P. perna *dominates the lowest mussel zone and *M. galloprovincialis *the highest, while the two species co-exist in the mid-zone [44]. Previous studies showed that partial habitat segregation of these two species results from a combination of biotic and physical conditions along a gradient of multiple stresses. *M. galloprovincialis *dominates the higher mussel zone, where *P. perna *is typically sparse, presumably because it has greater tolerance to emersion stress [45-47]. In the lower mussel zone, where hydrodynamic stress is higher, *P. perna *initially facilitates survival of *M. galloprovincialis *by providing protection against waves, but later it excludes the invasive species through interference competition for space [48]. In addition, recruitment failure (low settlement, high mortality) contributes to the exclusion of *P. perna *from the high shore, while the exclusion of *M. galloprovincialis *from the low mussel zone is likely to be by post-recruitment mortality [49], possibly strengthened by periodic heavy recruitment of *P. perna *[10].

This study reports differences in gaping behaviour of these two co-existing and competing mussel species, *M. galloprovincialis *and *P. perna*. We hypothesise that behaviour plays a key role in habitat segregation between the two species. Specifically we test the hypothesis that behavioural dissimilarity will expose the two species to different degrees of abiotic stress, forming the mechanism behind their different physiological tolerances in terms of desiccation resistance and Hsp protein expression. We predict that the non-gaping species will cope better with desiccation stress than the gaping species, but at the price of higher production of Hsp proteins.

## Methods

### Laboratory experiments

Mussels (shell length 4-5 cm) of *Mytilus galloprovincialis *and *Perna perna *were collected from the middle of the mussel zone in Plettenberg Bay on the south coast of South Africa (34° 22' S, 23° 22' E). Before the experiments, all individuals were acclimated in aerated seawater for 48 h in a controlled environment chamber at 18°C under a 12:12 h light: dark regime in 100 × 60 × 50 cm tanks containing 125l of seawater.

#### Gaping behaviour

Sixty mussels of each species, from each zone were exposed to air for 6 h in a controlled environment chamber at 17°C and at 37°C (humidity 60%). Percentage of gaping mussels and number of valve movements (per 6 h) were noted by visual observation, without recording the width of the gape. Independent measures were obtained by selecting 10 different mussels for each time (n = 10). The experiment was repeated 4 times.

#### Water loss

Ten individuals of each species, from each zone were exposed to air for 6 h in a controlled environment chamber at 17°C and 37°C (humidity 60%). Individuals were weighed (± 0.01 g) at the beginning of the experiment and every 20 minutes to record loss of water. At the end of the experiment, each animal was dried at 60°C to constant weight (including shell valves) and the dry weight (tissue and shell) was measured. The value obtained was used to calculate percentages of water loss.

#### Desiccation experiment

Mussels (n = 15 each species, from each zone) were exposed to air in a controlled environment chamber at 17°C and 60% humidity. Mussel mortality was assessed as failure to close the valves when disturbed. Mortality was checked every 24 h for 4 days and the experiment was repeated 4 times.

### Heat shock protein expression in the field

#### Preparation of gill tissue

Specimens were collected from Plettenberg Bay during low tide when the tidal level was at the lowest point of the day of collection. In the field, gill lamellae were dissected from *Mytilus galloprovincialis *from the high and mid mussel zones, and from *Perna perna *from the mid and low mussel zones (n = 6 each species, each zone). Approximately 100 mg of tissue was homogenized with a pipette tip in 350 μl of SDS/PAGE sampling buffer (0.5 M Tris-HCl, pH = 6.8, 10% glycerol, 10% SDS, 5% β-mercaptoethanol, 0.05% bromophenol blue). The homogenate was heated at 100°C for 4 minutes using a car kettle. In the laboratory, samples were centrifuged at 12000 g for 15 minutes at room temperature and the resulting supernatant was used for electrophoretic analysis.

#### Electrophoresis, immunodetection, and chemiluminescent autoradiography

Proteins were separated by SDS/PAGE [15% (w/v) gel; [50]] and transferred to nitrocellulose [[51]; purchased from Amersham Pharmacia Biotech (Piscataway, NJ, U. S. A.)]. A sample of a mouse fibroblast cell line (NIH 3T3) crude extract, expressing Hsc70 was included on each gel as a positive control and as an internal standard to allow comparison of multiple Western analyses. Proteins were revealed by chemiluminescent-based immunodetection (ECL Advance Western Blotting detection kit, Amersham) and digitally captured using a chemidoc (Biorad). The mouse monoclonal primary antibody [product no. SPA-820, purchased from StressGen Biotechnologis Corp. (Victoria, BC, Canada)] specific for Hsc70 and Hsp70 was diluted 1:5000 in Tris-buffered saline blocking solution [50 mMl Tris/HCl (pH 7.5)/150 mM NaCl/1% (w/v) non-fat milk powder]. The horseradish peroxidase-conjugated anti-mouse IgG secondary antibody (NA931, Amersham) was used at 1:5000 in the same blocking solution.

#### Image analysis and quantification of expression of heat-shock proteins

Digitised images were analysed with image analysis software [ImageJ Processing and Analysis; [52]] to quantify band intensities. All digital images were captured at a range of exposures, and only those images that were captured within the linear phase of the exposure were analysed for variation in Hsps levels. Separate Western blots were treated and exposed identically.

### Statistical analyses

Data were transformed to fulfil pre-requisites for parametric analysis (Cochran's Test) and were analysed using GMAV5 software. A nested design was used in order to assess differences between species across their vertical zonation and to compare the effects of zone only within each species.

Because *M. galloprovincialis *did not show any sign of gaping behaviour, only *P. perna *results were included in the analysis. Percentage of gaping mussels and total number of valve movements were analysed separately with a 3-way ANOVA with temperature, zone and time as fixed factors. Each run of the experiment produced a single value and was considered to be a replicate, giving n = 4 for each test.

At the end of the experiment, percentages of water loss were analysed with 3-way nested ANOVA, with species and temperature as fixed factors and zone nested in species.

Last measures of the desiccation experiments (4 replicates) were analysed for significant differences between the two species using 2-way nested ANOVA, with species as a fixed factor and zone as a nested factor.

Variation in Hsps was analysed using 2-way nested ANOVA with species as a fixed factor and zone nested in species.

## Results

### Laboratory experiments

#### Gaping behaviour

*Mytilus galloprovincialis *did not show gaping behaviour at either temperature. *Perna perna *exhibited gaping at both temperatures, with an increase in ventilation movements (number of gapes per hour) and of gaping individuals at the higher temperature (37°C; Table 1). While the behaviour was observed for only the first two hours at 17°C, 36% of mussels kept gaping until the 5^th ^hour at 37°C. Moreover, gaping behaviour did not vary significantly as a function of tidal height (3-way ANOVA, arcsine transformed p = 0.74 and p = 0.19, Table 2a,b), except for ventilation movements at 17°C during the first two hours (low zone < mid zone). Ventilation movements decreased with time at both temperatures (Table 2b).

**Table 1:** Gaping behaviour.

A				
	**17°C**		**37°C**	

**Time (h)**	**% gaping individual**	**Ventilation movements (h^-1^)**	**% gaping individual**	**Ventilation movements (h^-1^)**

1	100 (±0)	2.85 (±0)	100 (±0)	3.6 (±0.11)
2	92.5 (±9.57)	0.675 (±0.15)	100 (±0)	1.625 (±0.09)
3	0 (±0)	0 (±0)	87.5 (±5)	1.5 (±0.08)
4	0 (±0)	0 (±0)	67.5 (±20.6)	1.175 (±0.09)
5	0 (±0)	0 (±0)	35 (±12.9)	0.525 (±0.12)
6	0 (±0)	0 (±0)	0 (±0)	0 (±0)

**B**				

	**17°C**		**37°C**	

**Time (h)**	**% gaping individual**	**Ventilation movements (h^-1^)**	**% gaping individual**	**Ventilation movements (h^-1^)**

1	100 (±0)	2.825 (±0.09)	100 (±0)	3.55 (±0.13)
2	90 (±14.1)	0.675 (±0.15)	97.5 (±5)	1.6 (±0.22)
3	0 (±0)	0 (±0)	85 (±5.7)	1.425 (±0.05)
4	0 (±0)	0 (±0)	70 (±18.25)	1.125 (±0.05)
5	0 (±0)	0 (±0)	37.5 (±21.6)	0.575 (±0.22)
6	0 (±0)	0 (±0)	0 (±0)	0 (±0)

Gaping behaviour of *P. perna *from (A) low and (B) mid mussel zone at 17 and 37°C.

**Table 2:** Statistical results of gaping behaviour of *P. perna*.

A				
**Source**	**Df**	**MS**	**F**	**p**

Temperature	1	19744.7553	482.92	< 0.001
Zone	1	4.5052	0.11	0.7409
Time	5	20900.3874	511.19	< 0.001
TemperatureXZone	1	0.5867	0.01	0.9050
TemperatureXTime	5	3564.9672	87.19	< 0.001
ZoneXTime	5	8.7563	0.21	0.9554
TemperatureXZoneXTime	5	3.4142	0.08	0.9946
Res	72	40.8859		

**B**				

**Source**	**df**	**MS**	**F**	**p**

Temperature	1	4278321.4838	11.25	< 0.05
Zone	1	186102.4817	2.33	0.1878
Time	5	189657.2822	78.15	< 0.001
TemperatureXZone	1	186631.2067	2.13	0.2044
TemperatureXTime	5	380325.6385	15.67	< 0.001
ZoneXTime	5	80012.3514	3.30	< 0.01
TemperatureXZoneXTime	5	87699.6844	3.61	< 0.01
Res	72	24269.3795		

Results of 3-way ANOVA applied to (A) gaping individuals and (B) ventilation movements, with temperature, zone and time as fixed factors.

#### Water loss

**Figure 1:**
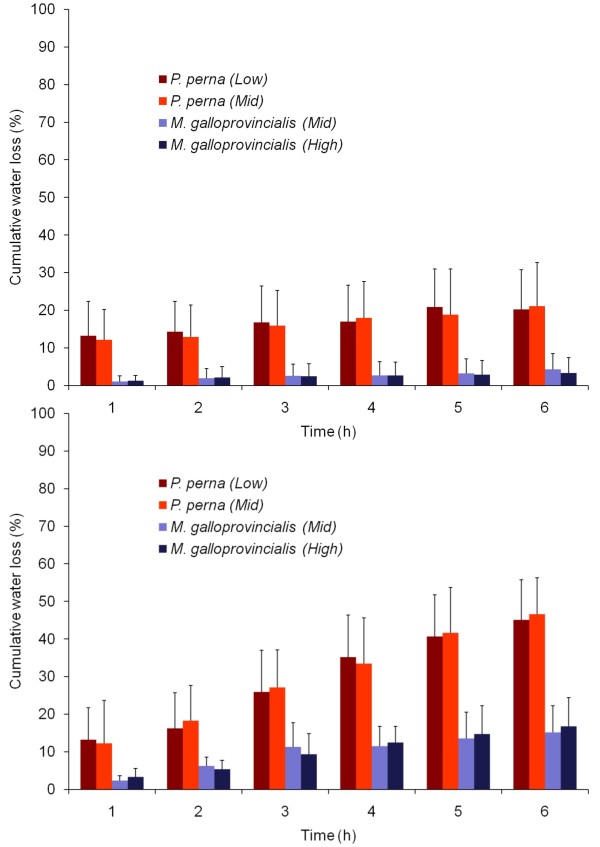
**Water loss**. Mean cumulative percentage of mussel water loss for 10 individuals of each species from each zone (+SD) when exposed to air at (A) 17°C, (B) 37°C.

While zone did not have an effect (3-way ANOVA, p = 0.98, Table 3; Figure 1a,b), water loss rates were higher for *P. perna *than for *M. galloprovincialis *and greater at 37°C than at 17°C for both species (p < 0.001). There was a significant species × temperature interaction (p < 0.01), reflecting the steeper water loss increase from 17 and 37°C for *P. perna*. Percentage of water loss increased every hour. After six hours at 17°C, *P. perna *and *M. galloprovincialis *had lost an average of 21% and 4% of total body water respectively, while at 37°C this percentage was 46% for *P. perna *and 16% for *M. galloprovincialis*.

**Table 3:** Statistical results of water loss.

Source	df	MS	F	p
Temperature	1	7414.4895	1949.35	< 0.001

Species	1	10815.9472	7213.70	< 0.001

Zone(Species)	2	1.4994	0.02	0.9785

TemperatureXSpecies	1	815.5941	214.43	< 0.01

TemperatureXZone(Species)	2	3.8036	0.06	0.9464

Res	72	68.9377		

Results of 3-way nested ANOVA applied to water loss, with temperature and species as fixed factors and zone nested in species.

#### Desiccation experiment

**Figure 2:**
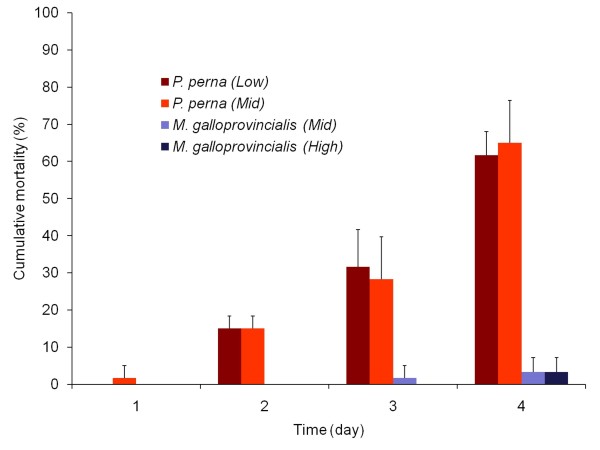
**Mortality when exposed to desiccation**. Mean cumulative percentage mortality for 4 replicates (+SD) each species from each zone when exposed to desiccation at 37°C.

Under desiccation stress, mortality rates were significantly higher for *P. perna *than for *M. galloprovincialis *(2-way ANOVA, p < 0.001, Table 4; Figure 2) but they did not show a zone effect (p = 0.8). *P. perna *from the mid and low zones began to die after 1 and 2 days respectively, while *M. galloprovincialis *from the mid and high zones began to die after 3 and 4 days respectively.

**Table 4:** Statistical results of desiccation.

Source	df	MS	F	p
Species	1	14400.0000	1296.00	< 0.001

Zone(Species)	2	11.1111	0.22	0.8040

Res	12	50.0000		

Results of 2-way ANOVA applied to desiccation, with species as a fixed factor and zone nested in species.

### Heat shock protein expression in the field

**Figure 3:**
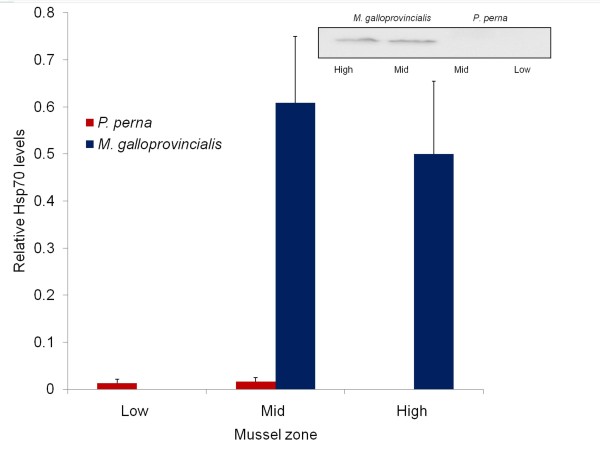
**Quantification of Hsp70 and example of Western-blot analysis**. Mean optical density quantification of Hsp70 (+SD) for six individuals each species from each tidal height, and example of Western-blot analysis. Note that low and high zone are dominated by one species only, *P. perna *and *M. galloprovincialis *respectively.

Levels of the 70-kDa molecular chaperones, Hsp70, and Hsc70 in mussel gill tissue were significantly higher in *M. galloprovincialis *than in *P. perna *(2-way ANOVA, p < 0.01, Table 5), but they did not vary significantly as a function of tidal height (p = 0.2; see Figure 3 for an example of Western analysis).

**Table 5:** Statistical results of Hsp70 quantifications.

Source	df	MS	F	p
Species	1	1.7480	98.57	< 0.01

Zone(Species)	2	0.0177	1.61	0.2254

Res	20	0.0110		

Results of 2-way ANOVA applied to Hsp70 quantifications, with species as a fixed factor and zone nested in species.

## Discussion and Conclusion

Invasive events appear to be highly idiosyncratic [53]. Our understanding of what allows some introduced species to become invasive, while others do not remain poor, likewise there is little consensus on what makes some ecosystems more vulnerable to invasion than others [54]. For example some studies indicate a link between species richness in the recipient community and the likelihood that it will be invaded Stachowicz et al. [55,56], while others do not [57,58]. Nevertheless, it is clear that the frequency of inoculation, or exposure to introduced species is important and this is part of the reason (perhaps the main reason) that coastal marine ecosystems suffer higher levels of biological invasions than almost any other ecosystem [20]. Yet even within these systems it is by no means clear why only some inoculations with alien species lead to successful invasions. In the past the focus has been on the differing physiological abilities of introduced species and their native competitors (e.g. [53]) and little attention has been paid to the effects of behaviour, partially because the behavioural repertoires of these species, especially the benthic taxa, are extremely limited. Our results indicate that even very simple behaviours can have profound effects, contributing to effective habitat partitioning, by altering the physical environment as actually experienced by competing species.

The rocky intertidal zone is among the most physically harsh environments on earth [21] and physiological adaptation to environmental stress (e.g. wave action, temperature and desiccation) plays a major role in determining potential patterns of vertical distribution (zonation). Nevertheless, species interactions (e.g. predation, competition and parasitism) modify this crude template profoundly [47,59,60] and behaviour in turn can affect both stress tolerance and species interactions [16,17,61]. We show that two competing intertidal mussels have clearly different gaping behaviour that strongly influences their tolerance of environmental stress, consequently playing a determining role in their distribution and the dynamics of co-existence between an invasive and an indigenous species. Non-gaping behaviour allows the invasive species to colonise the higher mussel zone that is incompatible with the gaping behavior of the indigenous mussel because of the high risk of desiccation. The greater tolerance of *Mytilus galloprovincialis *to desiccation comes at the price of a higher production of Hsps. When aerially exposed, *Perna perna *has significantly higher water loss than the non-gaping species, probably due to both evaporation and incidental expulsion of water during valve closure ([35], KRN and GIZ pers. obs.). This provides an explanation for the fact that it exhibits higher mortality rates than *M. galloprovincialis *when the two are transplanted to the higher shore [44,47]. These results are also in agreement with higher mortality rates of *P. perna *during air exposure at 17°C. In the field, the two mussel species are subjected to semidiurnal tides and they are usually exposed for less than six hours. The laboratory experiments lasted much longer than normal emersion, but produced mortality rates that presumably mirror sub-lethal fitness effects under field conditions where evaporative water loss is severe. Moreover, no intra-specific differences between mussels from different intertidal heights were observed in any of the parameters measured; this suggests that gaping behaviour alters water loss and desiccation but is related to species peculiarities rather than being a direct individual response to intertidal environmental conditions.

Previous studies have shown that, when kept in anoxic seawater, *M. galloprovincialis *and *P. perna *keep the valves open, but the latter is more sensitive to this condition and suffers higher mortality rates [62]. In addition, both species can regulate oxygen uptake down to concentrations of approximately 2-2.5 and 3.4 ppm respectively [63,64]. This is in accord with our results, indicating that during air exposure *P. perna *keeps the valves opened because of the need to rely on aerobic metabolism, while the invasive species has a higher tolerance of anoxic conditions and is able to maintain valve closure. Although it reduces desiccation rates, valve closure condemns mussels to a less efficient metabolism when catabolic, acidic end products have toxic effects and can accumulate to lethal levels [21,34,65,66]. Respiration rates of organisms usually increase with temperature and several studies have shown that mussels' oxygen consumption increases exponentially with temperature (e. g. [31,35]). Our results show that at 37°C mussels gape more than at lower temperatures suggesting that this behavior is linked to greater oxygen consumption, and supporting the important role of gaping in aerobic respiration [67]. In addition the greater ventilation rate seems to explain the higher water loss (through evaporation and/or active expulsion) observed at 37°C.

High levels of Hsps expression in intertidal organisms have been related to several stressors, including heat and anoxic stress [30,68]. The primary antibody used in this study is specific for both the inducible isoform, which is triggered by heat shock, and the constitutive isoform of Hsp70, which is affected by multiple physiological parameters. Previous studies found that there were higher levels of constitutive Hsp70 among high intertidal mussels in comparison to mussels from the low intertidal; in addition, constitutive Hsp70 levels in high intertidal bivalves were higher in summer than in winter [69,70]. This study shows that the expression of Hsps is significantly higher in *M. galloprovincialis *than in *P. perna*. Unexpectedly, we did not detect any intraspecific difference when comparing individuals from different heights of the mussel zone. This suggests that different expression levels of Hsps between *M. galloprovincialis *and *P. perna *are species-specific rather than being related to vertical stress gradient in the intertidal (but [68]). Several studies on different species have shown that gaping does not play a role in body temperature [19,35-37]; its most probable function is to allow aerobic respiration by maintaining an O_2 _gradient across the gills and the mantle wall [16,35,38]. This suggests that the higher stress experienced by *M. galloprovincialis*, as indicated by greater levels of Hsps expression, is related to anoxic stress rather than heat stress. Single stresses are rarely encountered in the natural environment. More typically, organisms encounter multiple stresses that interact with a variety of stress-responsive systems. In the case of sessile bivalves, a critical stressor that has been overlooked is simultaneous exposure to anoxia or hypoxia during periods of thermal stress. Since most periods of elevated ambient temperatures occur during emersion it will be important to address the question of whether anoxic and hypoxic conditions interact with heat-shock responses in intertidal bivalves.

Hawkins and Bayne [71] have estimated the cost of protein synthesis to constitute 20 - 25% of the energy budget of the mussel *Mytilus edulis*. This cost represents an additional energy burden because stress proteins do not directly contribute to growth or reproduction, and because under stress conditions Hsps may be synthesized preferentially, so that other proteins critical for the normal functioning of the organism are either synthesized at reduced rates or not synthesized at all. Furthermore, the function of stress proteins may require considerable ATP turnover; refolding of a protein may consume in excess of 100 ATP molecules [29,72,73]. Previous studies on intertidal mussels have shown that a trade-off exists between metabolically demanding processes such as attachment strength and gonad maturation [74]. In *P. perna *and *M. galloprovincialis*; peaks in attachment strength coincide with periods of relatively low gamete production for both species, suggesting that they cannot afford to invest simultaneously in both processes [75]. Energetic constraints can change spatially according to gradients of multiple physical factors and challenge co-existing species differently [76]. *P. perna *attachment strength is higher than that of *M. galloprovincialis*, while the latter has a greater reproductive output [77,78]. On the more wave exposed open coast, both species have to increase their attachment strength but, for the invader this comes at the cost of reduced reproductive output [78]. The costs of Hsps expression can also affect fertility/fecundity, energy budgeting, and survival through the alteration of cell functioning and high energy consumption [30]. The higher Hsps production of the invasive *M. galloprovincialis *could represent a competitive disadvantage, limiting its ability to survive on wave exposed shores where greater attachment strength is required.

The paradigm that assumes that lower and upper vertical distribution limits in the intertidal are solely set by biological and physical factors, respectively, has been challenged in the last few decades. Recent studies indicate that vertical zonation, and community dynamics in general, are largely driven by the interplay between environmental stress and species interactions [75-77]. Our two studied mussels show different tolerances to wave and sand stress, two of the main environmental factors affecting these intertidal communities. *P. perna *is more resistant to hydrodynamic stress than *M. galloprovincialis*. This explains the lower vertical limit of the invasive species and also explains the high mortality rates of *M. galloprovincialis *on wave exposed shores [17,62]. On the other hand, the invasive species is less vulnerable to sand action [77]. Sand tolerance does not have a relevant role in the vertical zonation of these species, but accounts for high mortality rates of *P. perna *during periods of high sand accumulation in mussel beds. Moreover, competition is at least in part influenced by physiological tolerance of physical factors. *M. galloprovincialis *survives well in the high zone, while transplanted *P. perna *dies [47]. Our results clearly underline the crucial role of behaviour. It limits desiccation of the invasive species and promotes the invader's success in the high mussel zone. On the other hand, *P. perna *is restricted by its gaping behaviour to the lower shore where it is also able to exclude the other species [48]. This agrees with the distribution of the invasive species on the west coast of South Africa, where *P. perna *is naturally absent and *M. galloprovincialis *extends its domain to the lower mussel zone.

Our results indicate that the outcomes of invasive/indigenous interactions are not necessarily solely affected by the environmental conditions prevailing in the invaded region, but rather by the conditions physiologically experienced by each species. Behaviour can dramatically moderate an organism's experience of environmental conditions and so dictate physiological reactions, and consequently the limits of tolerance limits to the stresses imposed by those conditions. As both indigenous species and invaders must respond to environmental variations, the difference in their responses can determine the success of the invader and how it interacts with native species [5,6]. Here we show that even the simple behaviour of sedentary benthic animals can explain the mechanisms behind observed patterns of distribution and the ability of an introduced species to become invasive.

## Authors' contributions

KRN and GIZ conceived and designed the study, carried out the field work, participated in the laboratory experiments and statistical analysis, and wrote the manuscript. CDM conceived the study, participated to in its design and coordination and helped to draft the manuscript. LS carried out laboratory experiments. SR performed the statistical analysis. GLB participated in experimental design and helped to draft the manuscript. All authors read and approved the final manuscript.
